# Evaluating the Insomnia Severity Index among South African first responders: evidence from classical test theory, Rasch, and Mokken analyses

**DOI:** 10.3389/frsle.2025.1635434

**Published:** 2025-09-23

**Authors:** Tyrone B. Pretorius, Anita Padmanabhanunni

**Affiliations:** Department of Psychology, University of the Western Cape, Cape Town, South Africa

**Keywords:** insomnia severity index, classical test theory, rasch analysis, mokken analysis, dimensionality, reliability, validity

## Abstract

**Background:**

Sleep is essential for physical health and psychological wellbeing, and insomnia is strongly associated with mental health difficulties, including depression, anxiety, and fatigue. Among first responders, the prevalence of insomnia is particularly high due to chronic exposure to stress, trauma, and irregular work hours.

**Aim:**

As part of a broader study focusing on the mental health of first responders in South Africa, the current study examined the psychometric properties of the Insomnia Severity Index from three different psychometric perspectives: classical test theory, Rasch analysis and Mokken scale analysis.

**Methods:**

Participants were first responders (*n* = 429) in the Western Cape province of South Africa and they included police officers (n = 309) and paramedics (n = 120). They completed the Insomnia Severity Index (ISI), the Patient Health Questionnaire-9, the Generalized Anxiety Disorder-7, and the Chalder Fatigue Questionnaire.

**Results:**

The three psychometric paradigms converged to confirm that the ISI measures a unidimensional scale. Furthermore, all three paradigms provided evidence for the construct validity of the ISI. In addition, classical test theory indices provided evidence for convergent and discriminant validity. Lastly, the correlations between insomnia as measured by the ISI and depression, anxiety, and fatigue provided evidence for concurrent validity.

**Conclusion:**

These findings affirm that the ISI is a stable and sound tool for assessing insomnia severity within the first responder population. The absence of measurement bias across gender and professional roles also enhances the practical utility of the ISI, as it ensures equitable assessment across subgroups within the first responder workforce. The ISI emerges from this study as a valuable resource for clinicians, researchers, and occupational health professionals working with South African first responders.

## 1 Introduction

Insomnia is a sleep disorder that is highly prevalent in the general population but it often remains underdiagnosed and untreated (Morin et al., 2023). It is characterized by difficulty initiating and/or maintaining sleep despite opportunities for sleep and is subjectively experienced as adversely affecting daytime functioning. Insomnia can be either acute, lasting for 3 days to a week, or it can be chronic and persist for at least 3 months (Vargas et al., 2020). Insomnia has consistently been associated with adverse physical health outcomes and is a risk factor for hypertension and cardiovascular disease owing to the elevation of cortisol levels arising from lack of sleep (Dean et al., 2023). Insomnia commonly co-occurs with mental health disorders such as anxiety and mood disorders. This has been ascribed to a bi-directional relationship between insomnia and these conditions. For instance, both generalized anxiety disorder and major depressive disorder are associated with persistent worrying, rumination about potential negative outcomes and frequent negative thoughts about oneself, the world and the future. These cognitive processes trigger autonomic arousal and emotional distress, which can impact sleep initiation and maintenance. Furthermore, it can lead to attentional bias toward internal and external cues that signal that sleep is being disrupted leading to an escalation in anxiety, which culminates in sleep deficits (Harvey, 2002; Tang et al., 2023).

Insomnia, in turn, can produce distress and adversely impact interpersonal and occupational functioning, which can aggravate psychological symptoms. Owing to the co-occurrence of insomnia and mental health disorders, interventions have typically prioritized treating the primary mental health condition, based on the assumption that the insomnia will remit once the underlying disorder is addressed. However, insomnia has been found to precede comorbid mental health disorders, aggravate the symptoms associated with them and persist despite effective treatment of the co-occurring condition (Morin et al., 2023). This has prompted the recognition of insomnia as an independent disorder, necessitating direct treatment and it has driven the development of instruments to accurately assess, diagnose and monitor sleep disturbances.

The Pittsburgh Sleep Quality Index (PSQI) and the Epworth Sleepiness Scale (ESS), for example, are frequently used instruments to assess sleep problems (Buysse et al., 2008; Salihu et al., 2022). However, these tools offer only general assessments of factors such as daytime sleepiness, sleep quality, and disturbances, and are not specifically designed to measure insomnia. The Insomnia Severity Index (ISI) is a prominent instrument for measuring insomnia severity and perceptions of the condition (Bastien et al., 2001). The ISI captures the diagnostic criteria for insomnia outlined in the Diagnostic and Statistical Manual of Mental Disorders (American Psychiatric Association, 2022). The ISI has been translated and validated into many languages including Korean (Cho et al., 2014), Portuguese (Clemente et al., 2017), Moroccan (Oneib et al., 2022), Spanish (Fernandez-Mendoza et al., 2012), Hausa (Salihu et al., 2022), and Persian (Sadeghniiat-Haghighi et al., 2014). These studies have confirmed that the ISI is a reliable instrument. The factor structure of the ISI has been examined in several studies, predominantly using Exploratory Factor Analysis (EFA) and Confirmatory Factor Analysis (CFA) and have provided mixed and inconsistent results.

The original validation study examining the psychometric properties of the ISI was conducted with a clinical population and employed EFA, which yielded a three-factor solution (Morin, 1993). This was supported by a study that used CFA in a non-clinical Spanish-speaking population (Fernandez-Mendoza et al., 2012). Subsequent research identified three distinct types of one-factor models and five different types of both two-factor and three-factor models. For instance, Gerber and colleagues validated the German version of the ISI among three independent samples and reported the scale was unidimensional (Gerber et al., 2016). Similarly, Dragioti and colleagues, analyzed the Swedish version of the ISI and found that a one factor solution had the best fit. Support for the two-factor solution has emerged from a range of studies including a validation study of the ISI in a sample of Korean university students (Lee and Kim, 2023) and research among Arabic patients diagnosed with chronic conditions (Al Maqbali et al., 2022). In contrast, the three-factor solution has been supported in research undertaken with Italian clinical patients diagnosed with insomnia (Castronovo et al., 2016) and Chinese undergraduate students (Lin et al., 2018). A systematic review and meta-analytic study on the structural validity of the ISI identified thirteen distinct models across the literature (Dilshad Manzar et al., 2021). The wide range of multifactorial models suggests that insomnia is composed of heterogeneous latent constructs that emerge from a common set of symptoms. This variability implies that individuals may experience and interpret insomnia symptoms in different ways, leading to diverse underlying factor structures. It also highlights the complexity of insomnia as a clinical phenomenon, where shared symptoms such as difficulty falling asleep, staying asleep, or experiencing non-restorative sleep, may cluster differently depending on the population studied, the context, or methodological approaches used.

The current study focused on South African first responders and examined the psychometric properties of the ISI from three different psychometric perspectives: Classical Test Theory (CTT), Rasch analysis and Mokken Scale Analysis (MSA). First responders are frontline workers responsible for emergency medical care. This group of professionals include emergency medical service personnel (e.g., paramedics and ambulance personnel), firefighters and law enforcement officials. Their primary responsibility is to provide rapid and often life-saving assistance in situations that range from medical crises and accidents to natural disasters, violent incidents, and other critical emergencies. Due to the nature of their work, first responders are frequently exposed to high-stress environments, traumatic events, and unpredictable scenarios (Arjmand et al., 2024).

Existing research has confirmed that insomnia is a highly prevalent condition among first responders. In their meta-analytical study on sleep disorders among first responders, Huang and colleagues reported a prevalence rate of 28% for insomnia (Huang et al., 2022). These sleep disturbances have been attributed to the demands of their occupation including irregular work shifts, frequent exposure to potentially traumatic events and the physical and emotional toll of responding to crisis situations. Recent evidence also supports fear of sleep as a central mechanism underlying insomnia among first responders and develops in response to certain post-traumatic stress disorder (PTSD) symptoms, including intrusive re-experiencing of trauma through nightmares (Reffi et al., 2023; Lebeaut et al., 2022). These fears drive maladaptive coping behaviors including avoidance of sleep or reluctance to sleep at night (Reffi et al., 2023; Werner et al., 2021). Chronic insomnia is associated with fatigue, mood disturbances, difficulties with alertness and concentration, cognitive impairment and reduced quality of life (Bard et al., 2023). Given the prevalence of insomnia among first responders and its serious implications for both physical and mental health, it remains essential to ensure that the tools used to assess this condition are psychometrically sound and contextually appropriate. Furthermore, existing research has highlighted a wide range of multifactorial models for the ISI, underscoring variability in how the instrument performs across different populations and settings (Dilshad Manzar et al., 2021). These inconsistencies point to the importance of examining the underlying structure and item functioning of the ISI in specific contexts rather than assuming universal applicability.

The ISI has been used in the South African context but the reporting of the psychometric properties of the scale has been limited. Bentley et al. (2025), for example, used the ISI to examine sleep deterioration in a retrospective study of the general population during the COVID-19 pandemic. The ISI has also been used to assess a sleep intervention among adolescents (Rossouw et al., 2024), sleep difficulties among youth (De Doncker and Mclean, 2022) and the impact of sleep, physical activity and sedentary behavior on symptoms of depression and anxiety before and during the COVID-19 pandemic (Lewis et al., 2021). The ISI has also been used to investigate fear of not being safe during sleep and its association with sleep quality among adults (Correia et al., 2024). However, the psychometric properties of the ISI were not reported. Phaswana-Mafuya et al. (2019) used two items of the ISI to measure insomnia symptoms among pregnant women and reported satisfactory reliability (α = 0. 82).

By employing three distinct psychometric perspectives, the current study aims to provide a more comprehensive and nuanced understanding of the instrument's performance. While CTT assesses the reliability and validity of the scale at a global level and provides information on internal consistency and factor structure, Rasch analysis provides insights into the functioning of individual items (Meijer et al., 1990). MSA is a nonparametric item response theory (IRT) method that further evaluates an instrument's scalability and monotonicity, which are important for determining whether items consistently reflect increasing levels of insomnia severity (Franco et al., 2022). Taken together, these analyses have the potential to provide robust evidence on the reliability, dimensionality, and construct validity of the ISI among South African first responders.

## 2 Materials and methods

### 2.1 Participants and procedure

Participants were first responders (*n* = 429) in the Western Cape province of South Africa and they included police officers (n = 309) and paramedics *(n* = 120). We constructed electronic versions of the instruments described in the Measures section using Google forms. With the permission of administrators of Facebook groups that consisted of first responders, we posted this electronic link together with an invitation to participate in the study on these Facebook sites. We also obtained permission from the South African Police Services (reference: 3/34/2, 27 June 2023) and the Western Cape Department of Health (reference: WC_202307_041, 15 September 2023) to conduct the study. These permissions allowed for research assistants to visit police stations and hospitals to access potential participants in person.

The sample was predominantly male (55%) and the first responders mainly worked in an urban area (92.3%). Fifty-one percent of the sample was married while 35.2% were single. Slightly less than half of the sample (49.7%) had a post-matric qualification and 49.2% indicated that they had a matric certificate. The mean age of the sample was 39 years (*SD* = 9.93) and the mean number of years working as a first responder was 13.24 years (*SD* = 9.65).

### 2.2 Measures

The current study was part of a broader study on the mental health of first responders in South Africa. In addition to the ISI, participants completed the Patient Health Questionnaire-9 (PHQ-9: Kroenke et al., 2001), the Generalized Anxiety Disorder-7 (GAD-7: Spitzer et al., 2006), and the Chalder Fatigue Questionnaire (CFQ: Chalder et al., 1993). The last three instruments were included for the purpose of establishing the concurrent validity of the ISI.

The ISI is a brief screening measure of insomnia and consists of seven items that are scored on a 5-point scale ranging from 0 to 4, where higher scores reflect more acute symptoms of insomnia. An example of an item of the ISI is “how noticeable to others do you think your sleeping problem is in terms of impairing the quality of your life?”. The initial validation study reported an internal consistency estimate of α = 0.74 for the scores of the ISI (Bastien et al., 2001). The authors also demonstrated that the ISI was sensitive enough to detect changes in insomnia resulting from treatment interventions. Although the ISI has previously been used in South Africa (eg., Correia et al., 2024), we could not find any study that reported on the reliability of ISI scores when used in the country.

The PHQ-9 is a nine-item measure that is used to screen for and diagnose depression. It is responded to on a four-point scale that ranges from “*not at all*” (0) to “*nearly every day*” (3), and higher scores on the PHQ-9 reflect higher levels of depression. An example of an item of the PHQ-9 is “over the last 2 weeks, how often have you been bothered by feeling tired or having little energy?” The initial validation study reported Cronbach's alphas of 0.89 and 0.86 for the scores of the PHQ-9 in two different studies and correlations with the Short-Form General Health Survey (Stewart et al., 1988) served as evidence for construct validity (Kroenke et al., 2001). The PHQ-9 has previously been used in South Africa and Cronbach's alphas greater than 0.70 have generally been reported (e.g., Bhana et al., 2015; Rakshasa-Loots et al., 2023; Kigozi, 2020).

The GAD-7 consists of seven items and is used to screen for generalized anxiety disorder. Responses to the seven items are made on a four-point scale that ranges from “*not at all*” (0) to “*nearly every day*” (3), and higher scores on the GAD-7 reflect higher levels of depression. An example of an item of the GAD-7 is “over the last 2 weeks, how often have you been bothered by being so restless that it is hard to sit still?” The authors of the GAD-7 (Spitzer et al., 2006) reported an estimate of internal consistency of 0.92 for the scores of the GAD-7 with patients in primary care facilities and also provided evidence of criterion, construct, and factorial validity. In South Africa, reported reliability estimates for the scores of GAD-7 were generally greater than 0.70 (e.g., Kigozi, 2021; Tadi et al., 2022).

The CFQ consists of 11 items and is used to measure the extent and severity of fatigue and general tiredness. It is scored on a four-point scale that ranges from “*less than usual*” (0) to “*much more than usual*” (3) and higher scores reflect greater fatigue. An example of an item of the CFQ is “do you have problems with tiredness?” The authors of the scale (Chalder et al., 1993) reported an estimate of internal consistency of 0.89 for the scores of the CFQ and factor as well as receiver operating curve analyses provided support for the validity of the CFQ. In South Africa, only one study was found that used the CFQ (Coetzee et al., 2018: α = 0.83).

### 2.3 Ethics

The study received ethical approval from the Humanities and Social Sciences Research Ethics Committee of the University of the Western Cape (ethics reference: HS23/2/4, May 23, 2023) and was conducted in accordance with the guidelines of the Declaration of Helsinki. Participants provided informed consent on the first page of the electronic link. Participation was voluntary and participants were informed on the landing page of the electronic survey that they may withdraw from the study at any time.

### 2.4 Analysis

CTT analyses were conducted with IBM SPSS for Windows version 30 and IBM SPSS Amos for Windows version 28 (IBM Corp., Armonk, NY, USA). Rasch analysis was conducted with Winsteps 5.8.0 (Linacre, 2023) and MSA with the package “Mokken” (Van Der Ark, 2012) in R software (R Development Core Team, 2020). There were no missing data as participants had to respond to every item of the survey.

#### 2.4.1 CTT indices

CTT indices obtained with SPSS included skewness and kurtosis, descriptive statistics (means and standard deviations), inter-item correlations, Item-Total Correlations (*ITC*), reliability indices (alpha, omega and composite reliability—*CR*), average variance extracted (*AVE*), maximum shared variance (*MSV*), average shared variance (*ASV*), and intercorrelations between variables (for concurrent validity). We used the indices of skewness and kurtosis to examine the distribution of data and in general data is regarded as normally distributed if skewness and kurtosis values ranged between −2 and +2 (SmartPLS, 2024). The internal consistency of the ISI was assessed using inter-item correlations, *ITC*, and the various reliability indices. Inter-item correlations should be between 0.15 and 0.85 and the average inter-item correlations should be between 0.15 and 0.50, while *ITC* should be greater than 0.50 (Hajjar, 2018; Paulsen and Brckalorenz, 2017). *ITC* that meet these criteria also contribute to the construct validity of the ISI since this would demonstrate that the items contribute to the measurement of the latent variable, insomnia (Devon et al., 2007). With respect to the reliability coefficients, it is recommended that an acceptable reliability coefficient for scales should be at least 0.70 for research purposes and much higher for clinical purposes (Devon et al., 2007). *AVE* refers to the amount of item variance extracted by the latent variable and if it is greater than 0.50 and less than *CR* it is indicative of convergent validity (Posch et al., 2019). *MSV* is the square of the correlation between insomnia and the variable it is most highly related to while *ASV* is the mean of the squared correlations between insomnia and all variables. Discriminant validity is established if *ASV* is greater than *MSV* and *ASV*, as this would indicate that the latent construct has more in common with the items that contribute to its measurement than with other related variables (Almén et al., 2018). With respect to concurrent validity we hypothesized, based on the available literature (Riemann et al., 2020; Taylor et al., 2005; Vethe et al., 2018), that insomnia would be positively associated with depression, anxiety, and fatigue.

#### 2.4.2 Factor structure and dimensionality

To examine the factor structure of the ISI, we conducted an EFA (principal components with varimax rotation) as well as a CFA. Prior to the EFA we used the Kaiser–Meyer–Olkin (KMO) measure of sampling adequacy and Bartlett's test of sphericity to determine whether the data is suitable for factor analysis. If Bartlett's test was significant and KMO was greater than 0.50, items would be considered sufficiently correlated to conduct factor analysis. When factor loadings (λ) in EFA are greater than 0.50 it also provides evidence of construct validity as it would indicate that the latent construct is well represented by the individual items (Hair et al., 2010). We used CFA in SPSS Amos to examine three models of the factor structure of the ISI, a one-factor model in which items load on a single factor, a correlated two-factor model in which items load on two factors (called severity and impact which are the subscales most commonly identified in the literature) that are correlated, and a bifactor model in which items load on a total scale (also called a general factor) as well as on the two subscales (also referred to as specific factors) of severity and impact. To examine the extent to which the models fit the data to an acceptable degree, the following are the minimum fit indices recommended (Gaskin et al., 2022): chi-squared (χ2: should ideally be non-significant), relative χ2 (χ2 divided by degrees of freedom: should be between 1 and 3), the comparative fit index (CFI: should be ≥0.95), the standardized root mean square residual (SRMR: should be ≤ 0.08), root mean square error of approximation (RMSEA: should be ≤ 0.06) and the p-value of close fit (PCLOSE: should be statistically non-significant i.e., *p* > 0.05). In addition to these recommended fit indices, we also included a model comparison index, Akaike's information criterion (AIC) and lower values of AIC indicate better models (Arbuckle, 2012) and an AIC difference (ΔAIC) between the baseline model and another model of greater than −2 reflects that the baseline model is the better model (Bevans, 2023).

In the case of the bifactor model we also used a freely available online Excel spreadsheet (Dueber, 2017) to obtain ancillary bifactor indices which clarifies whether the specific factors explained a sufficient amount of variance in the items over and above the variance explained by the general factor. The bifactor indices included explained common variance (*ECV*), omega hierarchical (*omegaH* for the general factor and *omegaHS* for specific factors) and the percentage of uncontaminated correlations (*PUC*). *ECV* is the percentage of variance explained by the general factor and the specific factors, respectively and an *ECV* greater than 0.70 for the general factor would indicate that the instrument is essentially unidimensional (Rodriguez et al., 2016). *OmegaH* is an estimate that represents the proportion of variance in observed scores that is due to the general factor. In the case of the specific factors, *omegaHS* refers to the proportion of systematic variance accounted for by a specific factor after the variance attributable to the general factor and other specific factors is controlled. An *omegaH* greater than 0.80 would indicate a very reliable factor (Schmitt et al., 2018). *PUC* refers to the percentage of correlations between items which reflect variance from the general factor and it is suggested that *PUC* should be used in conjunction with *ECV* and *omegaH* to draw conclusions about the dimensionality of an instrument. In this regard, Reise et al. (2013) suggested that when *PUC* is less than 0.80, *ECV* greater than 0.60, and *omegaH* greater than 0.80, any dimensionality that might exist is not strong enough to override an interpretation of the instrument in question as unidimensional.

To further examine the dimensionality of the ISI, we conducted parallel analysis using SPSS syntax that is freely available online (O'connor, 2000). Parallel analysis is regarded as one of the most accurate ways to determine the number of factors present in a set of items (Zwick and Velicer, 1986) and it involves comparing eigenvalues obtained in the current study with eigenvalues obtained in a number of simulated datasets (*n* = 1000). The number of eigenvalues obtained in the current dataset that are greater than the 95^th^ percentile of the simulated eigenvalues represent significant factors (Hayton et al., 2004). If only one eigenvalue is greater than the 95^th^ percentile of the simulated eigenvalues, the instrument in question should be regarded as unidimensional.

#### 2.4.3 Rasch analysis

The indices obtained in the Rasch analysis included infit and outfit mean square (*MnSq*) as well as item and person separation reliability and index and these indices contribute to assessing the construct validity of the ISI. Infit and outfit *MnSq* provide an indication of the extent to which items fit the Rasch model and values between 0.5 and 1.5 indicate good fit (Linacre, 2023). Person separation reliability and index provide an indication as to whether the scale can distinguish between low and high scorers on the latent variable and it is recommended that person separation index should be greater than 2 and person separation reliability greater than 0.80. Item separation reliability and index assess whether an item hierarchy exist and an item separation index greater than 3 together with an item separation reliability greater than 0.80 are recommended (Linacre, 2023). In Rasch analysis a principal component analysis (PCA) of the residuals (after the latent trait has been removed) is used to determine dimensionality. If an additional dimension, called the first contrast, has an eigenvalue greater than 2, it would be indicative of two or more items loading on a second factor, thus suggesting that an instrument is multidimensional. We also used Rasch analysis to determine measurement invariance across gender and across type of first responder using a Rasch index called Differential Item Functioning (*DIF*). *DIF* values greater than 0.50 would indicate that the item does not measure the same construct across groups (Linacre, 2023).

#### 2.4.4 Mokken analyses

In MSA, dimensionality is assessed using an algorithm called automated item selection procedure (AISP) which assigns a value of zero if an item is unscalable (does not load on a scale) and as many values as there are scales within a set of items. If all items are assigned the value of 1, it would indicate that all items load on a single scale (Van Der Ark, 2012). The strength of a scale is assessed by a scalability coefficient, *H* and an *H* value greater than 0.50 indicates a strong scale, while an *H* value less than 0.30 reflects a weak scale (Wind, 2017). MSA also provides an *H* value for each individual item (*H*_*i*_) which indicates the extent to which the item contributes to the measurement of the latent variable and *H*_*i*_ values should be greater than 0.30 (Mokken, 2011). An important assumption in MSA is monotonicity which implies that the probability of endorsing an item increases as the value of the latent variable increases. Violations of this assumption is indicated by the symbol #vi (the number of violations) and the index *Crit*—a *Crit* value greater than 80 indicates a significant violation of monotonicity (Sijtsma and Van Der Ark, 2017). Lastly, MSA also provides a Mokken reliability coefficient, *MS*_*rho*_.

## 3 Results

The inter-item correlations of the ISI as well as the CTT, Rasch and Mokken indices are reported in Table 1.

**Table 1:** Inter-item correlations, CTT, Rasch and Mokken indices for items of the ISI.

**Indices**	**1**	**2**	**3**	**4**	**5**	**6**	**7**
**Inter-item correlations**							
1. Difficulty falling asleep	—						
2. Difficulty staying asleep	0.72^***^	—					
3. Early morning awakenings	0.49^***^	0.53^***^	—				
4. Dissatisfaction with sleep	0.57^***^	0.53^***^	0.55^***^	—			
5. Sleep problems interfering	0.45^***^	0.44^***^	0.39^***^	0.42^***^	—		
6. Noticeability	0.40^***^	0.42^***^	0.43^***^	0.33^***^	0.61^***^	—	
7. Distress	0.57^***^	0.57^***^	0.49^***^	0.47^***^	0.62^***^	0.62^***^	—
**Classical test theory indices**							
Mean	1.43	1.54	1.65	1.85	1.66	1.39	1.37
*SD*	1.14	1.17	1.26	1.08	0.82	1.04	1.11
*ITC*	0.71^***^	0.71^***^	0.62^***^	0.63^***^	0.63^***^	0.59^***^	0.73^***^
λ	0.80^***^	0.80^***^	0.72^***^	0.73^***^	0.71^***^	0.82^***^	0.82^***^
**Rasch indices**							
Infit *MnSq*	0.94	0.96	1.33	1.01	0.78	1.11	0.88
Outfit *MnSq*	0.90	0.92	1.25	0.99	0.96	1.06	0.83
*DIF* across gender	0.00	0.00	−0.23	0.00	0.00	0.29	−0.08
*DIF* across type of first responder	−0.12	−0.31	0.46	0.08	0.17	−0.14	−0.16
**Mokken indices**							
AISP value	1	1	1	1	1	1	1
*H_*i*_*	0.57	0.57	0.52	0.54	0.54	0.49	0.59
*SE of H_*i*_*	0.03	0.03	0.03	0.03	0.03	0.03	0.03
*#vi*	0	0	0	0	0	0	0
*Crit* value for monotonicity	0	0	0	0	0	0	0

ITC, item-total correlation; MnSq, mean square; DIF, differential item functioning; AISP, automated item selection procedure; H_*i*_, scalability coefficient of individual items; SE, standard error; #vi, violations of monotonicity.

*** *p* < 0.001.

Table 1 shows that the inter-item correlations ranged between 0.33 and 0.72 and all were statistically significant (*p* < 0.001). The average inter-item correlation was 0.50. The item-total correlations ranged between 0.59 and 0.73 and were all statistically significant (p < 0.001). KMO was greater than 0.50 (0.86) and Bartlett's test was significant, indicating that the data was suitable for factor analysis. EFA (principal components with varimax rotation) extracted one factor which accounted for 57.68% of the variance. The factor loadings ranged between 0.71 and 0.82 and were all statistically significant (*p* < 0.001).

With respect to the Rasch indices, infit and outfit MnSq values were at an acceptable level and ranged between 0.78 and 1.33 for infit MnSq and between 0.83 and 1.25 for outfit MnSq. The items of the ISI demonstrated measurement invariance across gender (DIF = −0.23 to 0.29) and across type of first responder (DIF = −0.31 to 0.46).

With regards to the Mokken indices, Table 1 also shows that AISP found that all of the items of the ISI loaded on one scale as denoted by the value 1 for all items. Furthermore, the Hi coefficients for the individual items of the ISI were all above 0.30 and ranged between 0.49 and 0.59. There were no violations of monotonicity as #vi and Crit values were 0 for all items of the ISI.

**Figure 1:**
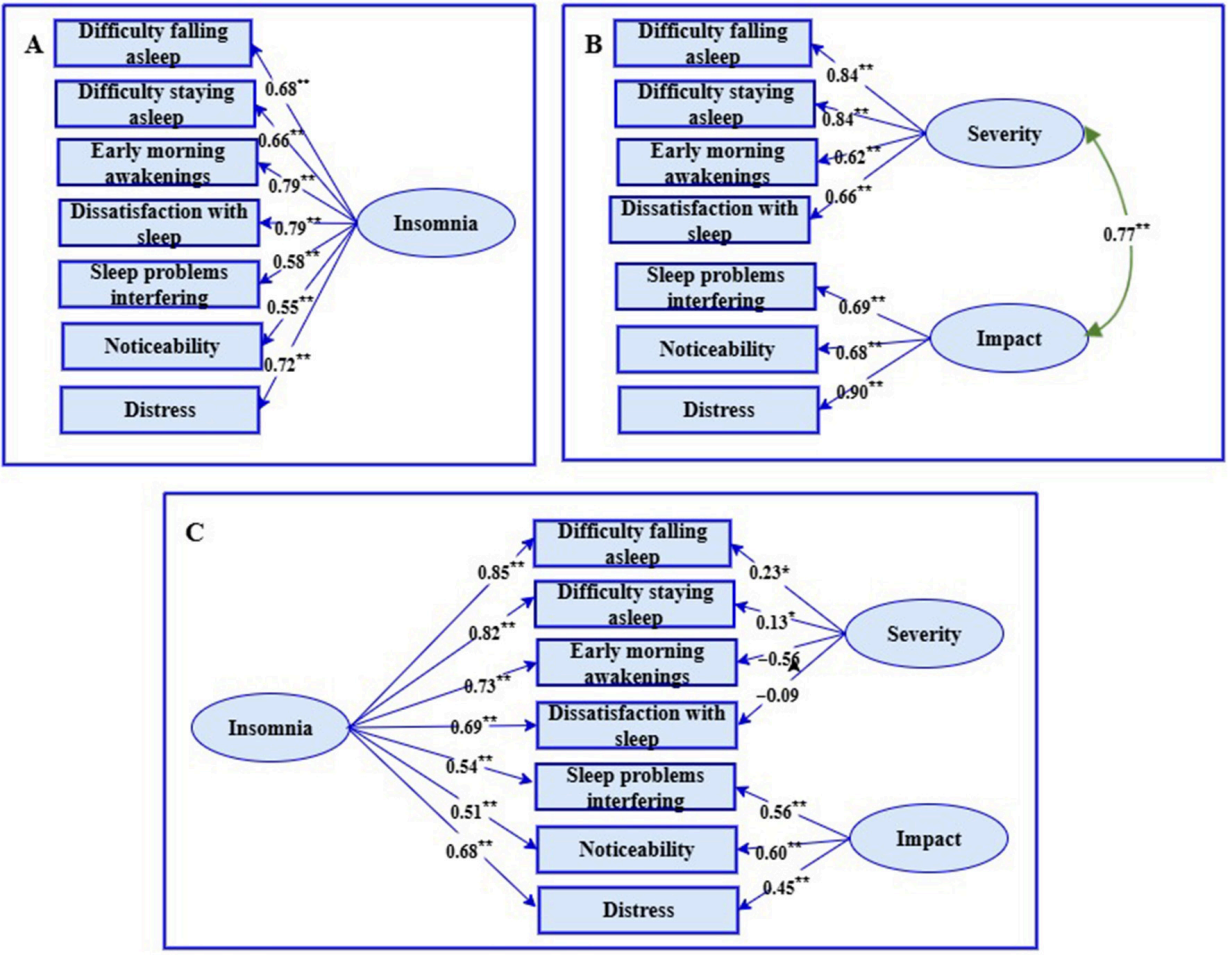
Three models of the factor structure of the insomnia severity index. **(A)** = one-factor model, Figure **(B)** = correlated two-factor model, Figure **(C)** = bifactor model. Rectangles are observed variables, ellipses are latent variables. All regression coefficients are standardized.

The three models of the factor structure of the ISI that were examined with CFA are shown in Figure 1, together with standardized factor loadings. Figures 1A–C depict the one-factor, correlated two-factor, and bifactor models, respectively.

The fit indices associated with the three models in Figure 1 are reported in Table 2.

**Table 2:** Fit indices for three models of the structure of the ISI.

**Fit index**	**Good fit indicator**	**Bifactor model**	**One-factor model**	**Correlated two-factor model**
**χ2(df)**	Non-significant	**15.85(7)**	**19.85(8)**	**29.55(11)**
*p*-value	Non-significant	0.03	0.01	0.002
Relative χ^2^	Between 1 and 3	2.26	2.48	2.68
CFI	≥0.95	0.99	0.99	0.99
SRMR	≤0.08	0.02	0.02	0.03
RMSEA	≤0.06	0.05	0.06	0.06
PCLOSE	*p* > 0.05	0.37	0.29	0.20
AIC	Lower values	71.85	73.85	77.55
ΔAIC	>−2	—	−2	−5.7

CFI, comparative fit index; SRMR, standardized root mean square residual; RMSEA, root mean square error of approximation; PCLOSE, p-value of close fit; AIC, Akaike information criterion; ΔAIC, change in AIC; AIC change is relative to the bifactor model.

The fit indices in Table 2 indicated that all three models fit the data to an acceptable degree (relative χ^2^ < 3, CFI > 0.95, SRMR < 0.08, RMSEA ≤ 0.06, PCLOSE non-significant). However, the model comparison index, AIC, indicated that both the bifactor model and the one-factor model (AIC difference was 2) were better fits than the correlated two-factor model (AIC difference between the bifactor model and the correlated two-factor model was 5.7). In addition, the two factors were substantially correlated (*r* = 0.77, *p* < 0.001). Also, while the bifactor model demonstrated an acceptable fit, Figure 1C shows that the regression coefficients of the severity subscale were very problematic since two of the items were very low (“Difficulty falling asleep” and “Difficulty staying asleep”) and two were negative (“Early morning awakenings” and “Dissatisfaction with sleep”). This is in stark contrast to the factor loadings associated with these four items on the same subscales in the correlated two-factor model where the factor loadings were greater than 0.60. Both factor loadings that were very low were statistically significant (i.e., 0.23 and 0.13). This is to be expected given a sample size of *n* = 429, but it is way below the accepted threshold of 0.50 for meaningful factor loadings.

Since CFA found that the bifactor structure fitted the data to an acceptable degree, ancillary bi-factor indices were calculated to determine whether the specific factors (two subscales) explained a sufficient amount of variance over and above that explained by the general factor (total scale). In this regard the bifactor indices indicated that the general factor, insomnia, explained 73% of the variance in the items (*ECV*) while the specific factors explained 27% of the variance (*ECV*_*severity*_ = 8.3%, *ECV*__*impac*_t_ = 18.7%). *OmegaH* for the general factor was 0.82, reflecting a very reliable general factor, while for the specific factors it was 0.01 and 0.39, respectively. Further, a *PUC* value of 0.57 together with an *ECV* of 0.73 and omegaH of 0.82 provides strong evidence that the ISI should be regarded as essentially unidimensional. Parallel analysis also extracted only one component with an eigenvalue (4.04) that was greater than the eigenvalue of the 95th percentile (1.25) of simulated eigenvalues. The eigenvalue of the second component (0.93) was less than the 95th percentile (1.15) of simulated eigenvalues.

The CTT, Rasch and Mokken indices at scale level for the unidimensional ISI are reported in Table 3.

**Table 3:** CTT, Rasch and Mokken indices at scale level for the ISI.

**Index**	**Value**	**Acceptable value/level**
**Classical test theory indices**		
Cronbach's alpha	0.87	>0.70
McDonald's omega	0.87	>0.70
Composite reliability	0.90	>0.70
Average variance extracted	0.58	>0.50
Maximum shared variance	0.38	< AVE
Average shared variance	0.31	< AVE
**Rasch indices**		
Item separation reliability	0.95	>0.080
Item separation index	4.39	>3
Person separation reliability	0.85	>0.80
Person separation index	2.34	>2
Eigenvalue of first contrast	1.99	< 2
**Mokken indices**		
*H*	0.55	>0.50
Mokken scale reliability	0.89	>0.70

*H* = scalability coefficient.

Table 3 indicates that the CTT, Rasch and Mokken indices for the unidimensional ISI were all at an acceptable level. In the first instance, the eigenvalue of the first contrast was less than 2. Secondly, all the estimates of internal consistency were greater than 0.70 (α and ω = 0.87, CR = 0.90, MSrho = 0.89). Thirdly, AVE was greater than 0.50 and MSV and ASV were less than AVE. The scalability coefficient in Mokken analysis was greater than 0.50 (H = 0.55). The item separation reliability and index in the Rasch analysis were greater than 0.80 and 3, respectively, while the person separation reliability and index were greater than 0.80 and 2, respectively.

The descriptive statistics for and the intercorrelations between insomnia and the other variables that were included for the purpose of establishing concurrent validity, are reported in Table 4.

**Table 4:** Descriptive statistics and inter correlations between study variables.

**Variable**	**1**	**2**	**3**	**4**
1. Insomnia	—			
2. Depression	0.52^**^	—		
3. Anxiety	0.49^**^	0.73^**^	—	
4. Fatigue	0.61^**^	0.49^**^	0.50^**^	—
Mean	10.89	9.51	7.73	14.40
*SD*	5.79	6.42	5.86	6.81
Skewness	0.18	0.37	0.38	0.12
Kurtosis	−0.72	−0.49	−0.81	−0.25

** *p* < 0.001.

The indices of skewness in Table 4 ranged between 0.12 and 0.38, and those of kurtosis between −0.81 and −0.25. The values of skewness and kurtosis were thus within the acceptable range of −2 to +2. Table 4 also indicates that insomnia was significantly positively related to depression (*r* = 0.52, *p* < 0.001, large effect size), anxiety (*r* = 0.49, *p* < 0.001, medium effect size), and fatigue (*r* = 0.61, *p* < 0.001, large effect size). The positive relationships would indicate that higher levels of insomnia were associated with higher levels of depression, anxiety, and fatigue. The PHQ-9 contains one item related to sleep difficulty and to ensure that the correlation between depression and insomnia was not excessively influenced by this one item, we also examined the correlation between the two variables without this one item. The correlation remained a large effect (*r* = 0.50, *p* < 0.001 and the reliability of the PHQ-9 was not significantly affected (α = 0.88).

## 4 Discussion

This study aimed to evaluate the psychometric properties of the ISI among South African first responders, a population uniquely vulnerable to sleep disturbances due to the demanding and stressful nature of their work. Drawing on CTT, Rasch analysis and MSA, the study aimed to provide a comprehensive assessment of the instrument's reliability, validity and dimensional structure within this context.

Overall, the psychometric properties of the ISI, inclusive of reliability and validity, can be regarded as highly satisfactory. In the first instance, evidence from the three different psychometric paradigms overwhelmingly favored a unidimensional interpretation of the ISI, a finding that supports the one-factor solution reported in other studies (e.g., Gerber et al., 2016; Kaufmann et al., 2019). EFA extracted one factor and CFA fit indices found that a one-factor model and a bifactor model fit the data to an acceptable degree. However, ancillary bifactor indices found that the majority of the item variance was explained by a very reliable general factor. The unidimensionality of the ISI was confirmed with parallel analysis in that only one eigenvalue in the current dataset was greater than the 95th percentile of simulated eigenvalues. Further, a PCA of the residuals in Rasch analysis confirmed a unidimensional solution and AISP in Mokken analysis showed that all of the items of the ISI loaded on a single scale. The scalability coefficient in Mokken analysis, *H* was greater than.50 indicating a very strong unidimensional scale.

All the estimates of the reliability of the scores of the ISI may be considered satisfactory. The CTT reliability indices, alpha, omega and *CR* as well as the MSA index, *Ms*_*rho*_ exceeded 0.80 and was therefore well above the recommended threshold of 0.70 (Devon et al., 2007).

All three psychometric paradigms provided evidence for the construct validity of the ISI. In CTT the inter-item correlations, item-total correlations and factor loadings were within the ranges recommended in the literature (Hajjar, 2018; Devon et al., 2007; Paulsen and Brckalorenz, 2017; Hair et al., 2010) thus demonstrating that all the individual items of the ISI contribute to the measurement of the latent variable, insomnia. In Rasch analysis infit and outfit *MnSq* values were within an acceptable range (Linacre, 2023), thus confirming that all the items of the ISI fit the Rasch model. The person separation index and reliability as well as the item separation index and reliability confirmed that the ISI can distinguish between low and high scorers on the latent variable, and that an item hierarchy exists. *DIF* in Rasch analysis confirmed the measurement invariance across gender and type of first responder, thus indicating that the items of the ISI measure the same construct in men and women as well as in police officers and paramedics. Lastly, in MSA the scalability coefficients for the individual items of the ISI, *H*_*i*_, exceeded the threshold identified in the literature (Mokken, 2011), and these scalability coefficients, similar to item-total correlations, confirmed that all the items of the ISI contributed to the measurement of insomnia and that the latent construct of insomnia was well represented by its indicators.

CTT analysis also provided evidence for other forms of construct validity. AVE was greater than 0.50 and less than CR and according to Posch et al. (2019) this serves as evidence for convergent validity. AVE was greater than MSV and ASV and since this would indicate that the latent construct had more in common with the items that contributed to its measurement than with other related variables it provides evidence for discriminant validity. Lastly the strong correlations between insomnia as measured by the ISI and depression, anxiety, and fatigue provides evidence for the concurrent validity of the ISI.

The findings of this study offer important theoretical and practical implications for the assessment of insomnia, particularly within high-risk occupational groups such as first responders. From a theoretical perspective, the results reinforce and extend the existing understanding of the latent structure of insomnia as measured by the ISI. Evidence from all three psychometric paradigms favored a strong unidimensional solution, thus supporting the conceptualization of insomnia as a unified construct rather than a multidimensional syndrome in this population. This suggests that despite symptom diversity, the experience of insomnia can be meaningfully captured along a single underlying dimension. These findings affirm that the ISI is a stable and theoretically coherent tool for assessing insomnia severity across diverse groups within the first responder population. In terms of practical implications, the findings support the use of the ISI as a screening and diagnostic tool for insomnia among South African first responders. Its unidimensional structure simplifies scoring and interpretation, making it suitable for routine clinical use, as well as for incorporation into mental health surveillance and employee wellness programs. The ISI can assist in the early identification of individuals at risk, facilitate timely referrals, and guide the implementation of evidence-based interventions to mitigate the impact of sleep disturbances on overall wellbeing and job performance. The absence of measurement bias across gender and professional roles (e.g., paramedics vs. police officers) also enhances the practical utility of the ISI, as it ensures equitable assessment across subgroups within the first responder workforce.

While the findings of this study provide strong support for the unidimensionality, reliability and validity of the ISI when used with South African first responders, several limitations should be acknowledged. The study employed a cross-sectional design, which limits the ability to draw conclusions about the temporal stability of the ISI scores. Longitudinal research is needed to assess test–retest reliability and to evaluate how insomnia symptoms and ISI scores may change over time, particularly in response to interventions or varying occupational stressors. All data, including responses to the ISI and other psychological scales, were based on self-report. While self-report is a widely accepted method in research, it is susceptible to response biases such as social desirability, recall bias, or underreporting of symptoms due to stigma, especially within occupational groups like first responders. Although the study included a diverse sample of first responders (e.g., paramedics and police officers), the findings may not be generalizable to all emergency service personnel across South Africa or to first responders in other low- and middle-income countries. Furthermore, the use of the same subject samples for EFA and CFA represents another limitation. This approach may lead to overfitting and inflated estimates of model fit. Future research is needed to address these limitations by utilizing separate samples for EFA and CFA to enhance the validity and generalizability of the findings. Finally, the survey was administered online, which limited the ability to fully validate the population. The online format, while providing broad accessibility, restricted the ability to track those recruited from specific locations. Future studies could address this limitation by using more controlled recruitment methods to enhance population validation.

## 5 Conclusion

This study provides comprehensive evidence for the reliability and validity of the ISI among South African first responders, using three complementary psychometric approaches, namely CTT, Rasch and Moken analyses. The ISI had a unidimensional structure across all methods, which suggests that the instrument effectively captures the core features of insomnia as a single latent construct in this high-risk population. Furthermore, the instrument demonstrated strong internal consistency, item functioning, and measurement invariance across gender and professional subgroups, indicating that it is both psychometrically robust and fair. Given the high prevalence of insomnia and its impact on the physical and psychological functioning and occupational performance of first responders, the availability of a validated, contextually appropriate instrument remains essential. The ISI emerges from this study as a valuable resource for clinicians, researchers, and occupational health professionals working with South African first responders.

## Data Availability

The raw data supporting the conclusions of this article will be made available by the authors, without undue reservation.
